# Marginal Discrepancy of Cast Copings to Abutments with Three Different Luting Agents

**DOI:** 10.1155/2019/8657582

**Published:** 2019-09-02

**Authors:** José Rosas, Frank Mayta-Tovalino, Maria Eugenia Guerrero, Pedro Luis Tinedo-López, Claudia Delgado, Vanessa Z. Ccahuana-Vasquez

**Affiliations:** ^1^School of Stomatology, Universidad Privada San Juan Bautista, Lima, Peru; ^2^Faculty of Dentistry, Postgraduate Department, Universidad Peruana Cayetano Heredia, Lima, Peru; ^3^Department of Oral Implantology, Universidad Peruana Cayetano Heredia, Lima, Peru

## Abstract

**Aim:**

The purpose of this study was to evaluate the MD (marginal discrepancy) on the calcinable copings in abutments for cemented prostheses with three luting agents.

**Methods:**

Sixty-four analogs of CeraOne-type abutments (NACONIH code, Titanium Fix Implant Sytem SP, Brazil) were divided into four groups (*n* = 16). The copings were cast and placed on the CeraOne abutment analogs and cemented with eugenol-free zinc oxide (EfZO) (*n* = 16), with glass ionomer (GI) (*n* = 16), and with zinc phosphate (ZP) (*n* = 16), and as a control group, there were CeraOne plastic copings (NACOC code, Titanium Fix Implant Sytem SP, Brazil) (*n* = 16) which were not cemented with any material. After 24 hours, the MD of the four groups was measured. MD was evaluated using a stereoscopic microscope (Leica EZ4 W, Leica Microsystems, Germany) with an increase of ×100. MD was measured at four predetermined and equidistant sites with respect to the marginal line of the cast adaptation. The measurement was made from the distance between the free edge of the cast cylinder and the margin of preparation of the titanium abutment, with a level of statistical significance of *p* < 0.05.

**Results:**

Of the three fixing agents, the ZP was found to have the highest MD (53.59 ± 14.21 *μ*m); however, the lowest MD (41.72 ± 9.10 *μ*m) was found in the GI group. These differences are statistically significant at *p* < 0.001.

**Conclusions:**

In summary, according to our results, it was found that ZP cement showed the highest MD after cementation, followed by the glass ionomer, while EfZO showed the lowest MD.

## 1. Introduction

One of the most significant prerequisites for the long-term success of implant-supported prostheses lies on retention, resistance, and marginal sealing evaluation. Nowadays, it is known that cements exert more influence on resistance than on retention, and their function is to increase friction between prostheses retained by the implant and the abutment. Cement selection is one of the most important factors for determining the amount of retention attained on cement-retained restorations [1]. The selected cement can be either permanent or provisional, and a certain type of cement can be chosen based on the clinical situation [2]. The concept of using provisional cementation is to achieve restoration retrievability without endangering the implant restoration components.

There is scientific literature that has shown that the best fit accuracy has been found for screwed prostheses retained by implants compared to cemented prostheses retained by implants [3]. It is also known that cemented prostheses retained by implants are very aesthetic and do not have an occlusal orifice access that makes the occlusal surface more stable [4]. However, when the implant platform is at a very apical level and involves the prosthetic-abutment interface, the removal of the cement remnant is very difficult and may contraindicate the use of cemented prostheses retained by implants [5]. On the contrary, the selected fixing agent can improve the sealing of the space or can create conditions for a greater accumulation of microbial plaque, either by the degree of dissolution suffered by the humid environment or by the increase in space that depends on the thickness of the luting agent [6]. It should also be considered that if the cement widens the gap, once the restoration is cemented, occlusal corrections will be required that may alter the definitive restoration [7].

The biggest complication in the cementing process is the incomplete settlement on their respective abutments. This factor can decrease the retention and increase the marginal discrepancy (MD) or gap, which in turn favours the dissolution of the cement itself and the appearance of occlusal and periodontal alterations and, ultimately, may trigger the failure of the restoration. Other studies reported high levels of mucositis around dental implants with a discrepancy of marginal settlement or gap. The greater the gap between the restoration and the prosthetic abutment, the greater the dissolution and plaque adhesion, resulting in the growth of an inflammatory lesion in the mucosa next to the dental implant [8]. Finally, this would result in a greater crestal bone resorption [9]. Although accuracy of fit has a considerable effect on the clinical success of the cement-retained implant prostheses [10], to date no studies are available with regard to the marginal fit using different luting agents.

The purpose of this study was to evaluate the MD on cast copings fit over abutments cemented with three luting agents to reject the null hypothesis that refers to the fact that there is no statistical difference between the MD that occurs when using these cements.

## 2. Materials and Methods

### 2.1. Specimen Preparation

The research was an experimental in vitro design where sixty-four CeraOne abutment analogs (NACONIH code, Titanium Fix Implant Sytem SP, Brazil) were distributed into four groups (*n* = 16). Cast copings with CeraOne abutment analogs were cemented with eugenol-free zinc oxide (EfZO) (*n* = 16), with glass ionomer (GI) (*n* = 16), and with zinc phosphate (ZP) (*n* = 16). Sixteen CeraOne plastic copings (NACOC code, Titanium Fix Implant Sytem SP, Brazil) were not cemented and used as the control group. To facilitate manoeuvrability, all samples were embedded in acrylic resin based on previous studies [11, 12].

### 2.2. Casting of Abutments

CeraOne abutment analogs (code NACONIH, Titanium Fix Sytem SP, Brazil) were used. The metal caps were cast with a metal alloy Co-Cr (Metalloy CC Germany). The castings were then sprayed with a 5 mm diameter wax wire (wax wire for casting, Dentaurum, Germany) and invested in phosphate-bonded investment (Hi‐Temp, Whip Mix Co. Louisville, KY, USA). A loop was added to the occlusal surface of each cap with a wax trough (no. 10) to allow for future tensile testing. Finally, all the caps were cast with the lost wax method by the dental technician specializing in the Prosthesis Laboratory of the Universidad Peruana Cayetano Heredia (UPCH).

### 2.3. Sandblasting of Cast Copings

The castings were separated from the sprue with a carborundum disk and sandblasted, and their external surface refined slightly to produce intimate contact with the fixtures of the universal testing machine. The intaglio of the copings was sandblasted with 50 *μ*m aluminum oxide particles (Ivoclar Vivadent). Before cementation, the abutments and copings were cleaned of impurities with distilled water for 15 min in an ultrasonic bath. The intaglio was examined for irregularities with a stereomicroscope (Leica Microsystems LAS EZ version 2.0.0 Switzerland) at ×80 magnification.

Three different types of luting agents such as EfZO (Rely XTMTemp NE, 3M ESPE, Mexico), GI cement (GC Gold Label, GC Corporation, Japan), and ZP (DeTrey® Zinc, Dentsply DeTrey GmbH, Germany) were used. Each cement was used according to the manufacturer's instructions. For the EfZO group, a ratio of 3 : 1 powder-liquid was used that was mixed vigorously until achieving a homogeneous consistency that allows a correct cementation. For example, the ZP was prepared on a cold tile to avoid its exothermic effect by vigorous spatulation for 1 minute and 30 seconds in quantities of 2 mg of liquid and 1 gram of powder. Then, the cement was immediately taken to the work area to ensure adequate settlement of the metal cap. For the GI group, the powder and liquid were placed on a mixing paper. The powder/liquid ratio was 1.25 g of powder to 1.0 g of liquid. Subsequently, the powder was separated into 4 equal parts and was mixed one by one with the liquid for 40 seconds avoiding contact with water at all times.

The copings for all specimens were cemented with a standardized force of 49 N applied along the axis of the abutment pair within 10 min, according to American National Standards Institute/American Dental Association Specification No. 96 [13]. Finally, all specimens were stored for 24 h in distilled water at 37°C. All cast copings were cemented by digital pressure by a calibrated operator.

### 2.4. Marginal Discrepancy

**Figure 1:**
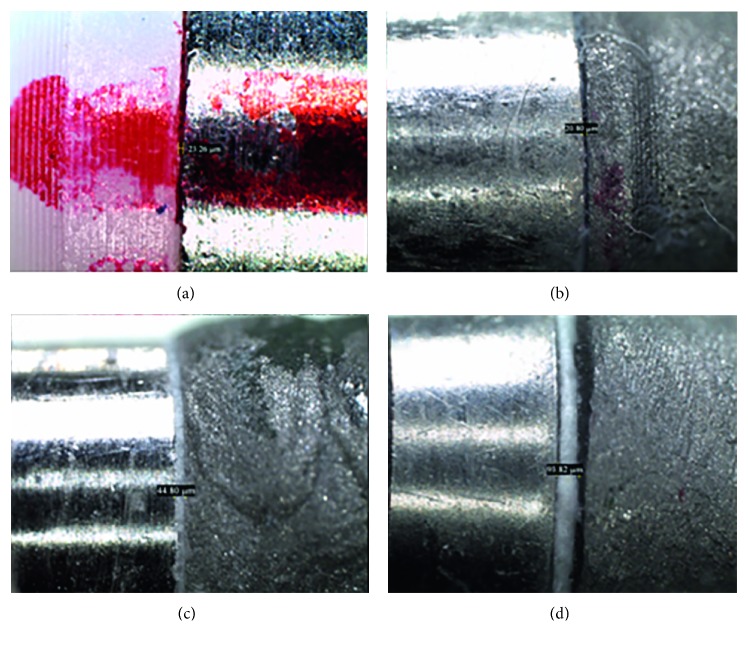
Marginal discrepancy measurements analyzed with the stereomicroscope. (a) Plastic copings without cementation positioned on the implant abutment analog. (b) Cast copings cemented over CeraOne abutment analogs using eugenol‐free zinc oxide. (c) Cast copings cemented over CeraOne abutment analogs using glass ionomer. (d) Cast copings cemented over CeraOne abutment analogs using ZP.

After 24 hours of cementation, MD of the four groups was measured. According to different studies [1–10] that used the same measuring instrument, it was decided to evaluate MD using a stereoscopic microscope (Leica EZ4 W, Leica Microsystems, Germany) with an increase of ×100. MD was measured at four predetermined and equidistant sites corresponding to the faces (mesial, distal, vestibular, and palatine) for each sample along the marginal termination line to have a more representative and standardized measurement. The measurement was made from the distance between the free edge of the cylinder and the margin of preparation of the titanium abutment. Then, a global average was obtained among the four mentioned measurements having a single value expressed in micrometers for each specimen evaluated. Finally, the images were obtained, recorded, and processed through the OmniMet™ modular imaging system (Buehler, Lake Bluff, Illinois, USA) from the Pathology Laboratory of UPCH (Figure 1).

### 2.5. Statistical Analysis

All analyses were performed in the SAS software package (version 9.0; SAS Institute, Cary, NC, USA) at a significance level of 5%. Normality of the error distribution and the degree of nonconstant variance were checked for each response variable. The results were averaged (mean + standard deviation) for each parameter. The Shapiro–Wilk test was used to determine the normal distribution. The Kruskal–Wallis test was applied to find out the significant difference between the study groups followed by the Bonferroni post hoc test. In all the above tests, *p* ≤ 0.05 was considered to be statistically significant.

## 3. Results

### 3.1. Comparison Significance of the Marginal Discrepancy

**Figure 2:**
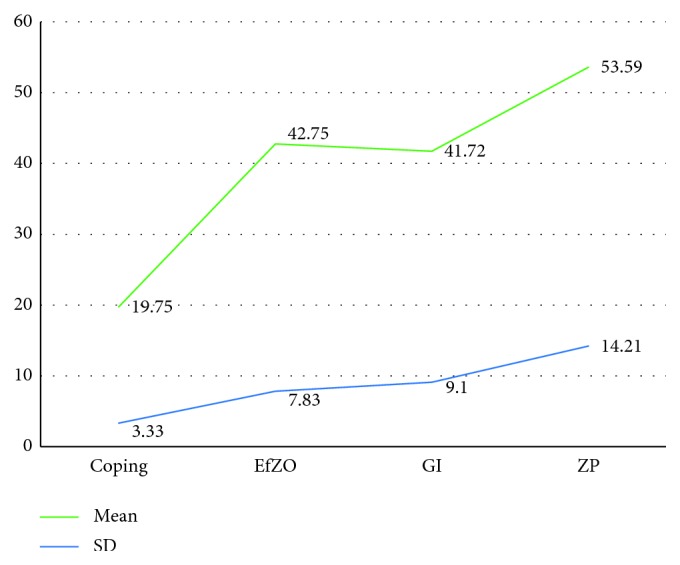
Comparison of the marginal discrepancy (*μ*) from the four groups. Coping, control group without any cement; EfZO, eugenol‐free zinc oxide; GI, glass ionomer; ZP, zinc phosphate.

To evaluate the DM that occurs at the time of melting the metal alloy for the manufacture of the caps on the dental implants, a total of 64 structures were manufactured, 16 of each group. The samples were prepared for the evaluation of the stereomicroscope where the descriptive statistics of the MD (Figure 2) for the groups showed average values that are summarized in coping (control group without any cement) 19.75 ± 3.33; EfZO, 42.75 ± 7.83; GI, 41.72 ± 9.10; and ZP, 53.59 ± 14.21 *μ*, respectively, in Table 1.

Only the ZP group did not achieve a normal distribution. The Kruskal–Wallis test revealed a statistically significant difference between the MD from the four groups. The Bonferroni post hoc test revealed statistically significant differences (*p* < 0.000) for all the groups unless between the EfZO group and the GI group (Table 2).

## 4. Discussion

The marginal accuracy of the cemented crown is of clinical importance and influences long-term survival of the implant retained prostheses. In this study, the parameter used for the measurement of the marginal misfit was absolute marginal discrepancy. According to some studies, the consideration for measurement of absolute marginal discrepancy should be measured from the margin of the cylinder casting to the titanium abutment preparation. Several possible differences in terms of materials used, the methodology followed for coping manufacture, the measurement instrument selected, the limits taken as a reference to quantify the MD, and even the discrepancy concept itself, which varies from one author to another, were taken into account when comparing our results with those of other authors. This lack of standardization on MD study protocols is undoubtedly one of the main causes of the disparity between the results published on this topic [14–20].

Depending on the type of alloy used, some studies compared the structure discrepancies considering different types of alloy. In this study, the dental alloy used was cobalt chromium, which despite presenting major disagreements, compared to other noble metals, no significant differences have been found [20–26].

The MD values showed statistically significant differences on the marginal settlement discrepancy after cementation using different cements (EfZO, GI, and ZP). It was also found that the groups that presented the lowest MD when using a standardized cementation force were in the following order EfZO and GI. When both cements were compared, no statistically significant differences were reported between these groups. The highest value for MD was reported for the ZP group. These results showed that it is important to take into account the type of cementing agent used to not alter the discrepancy after cementation. This MD variation can be partially explained by the different film thickness values of cementation materials that can range from 25 to 100 *μ*m [14, 15].

In 2008, Siadat et al. [16] evaluated the marginal discrepancy that occurs in 3 foundry techniques for the manufacture of metal structures on dental implants, for which they manufactured a total of 24 structures. Then, the samples were sectioned and prepared for the evaluation of the scanning electron microscope. A total of 24 frameworks were fabricated, 8 each with the burnout cap, with impression cap, and with the conventional wax-up technique. Specimens were sectioned and prepared for scanning electron microscope evaluation. The vertical discrepancy measurements for the 3 groups showed mean values of 53.74 ± 11 *μ*m, 63.6 ± 13.2 *μ*m, and 50.1 ± 17.3 *μ*m, respectively. The interfacial gap differences were not statistically significant for all groups (*P* > 0.05). In our study, we found a mean vertical discrepancy of 19.75 *μ* for the plastic copings. This value was raised to 53.59 *μ* once it was burnout and cemented with ZP at constant pressure.

In the dental literature, a similar measurement method to our study was performed by Wolfart et al. [17]. However, they also used a stereomicroscope and a similar computer support to the one this study developed. Four zones in each abutment were selected, and a series of MD measurements were made in each of them following equidistant reference lines established by the developed system. However, they used both sandblasted abutments and nonsandblasted abutments, with no differences in the MD in both techniques. In this research, nonsandblasted abutments were used and no statistically significant differences were found for the MD between the EfZO and the glass ionomer. This was contrary to what Wolfart et al. [17] published, which did find statistically significant differences between the EfZO cement and the GI. This could be due to nonabutment sandblasting. Our study obtained similar results, with a greater marginal discrepancy for the ZP cement.

Sutherland et al. [18] determined the mean MD of all-ceramic crowns cemented on implant abutments on the following groups: all-ceramic caps, all-ceramic crowns, and all-ceramic crowns cemented with ZP cement. The marginal fit in all-ceramic crowns cemented with ZP cement was 168.8 ± 23 *μ*m. Subgingival marginal discrepancies of the magnitude measured in this study have been shown to cause periodontal problems. In this study, a maximum value of 85.66 *μ* was obtained when the specimens were cemented with ZP.

The main difference of this study with others is that CeraOne abutments were used, which are clearly parallel to each other; therefore, the need arose to know what the impact of the cementing agents on the cementing of metal coping in pillars is with these characteristics since most published research carries out evaluations on divergent wall abutments.

On the one hand, despite our effort to achieve a homogeneous sample and control external variables, it is not possible to state with complete certify that the differences found in the groups are due exclusively to the characteristics of the cement. On the other hand, there are many other variables that could influence the results. For instance, we believe that it is difficult for one observer to execute the entire laboratory process with absolute precision on all occasions. So we think that a certain bias in the results is inevitable. However, we consider methodologically the fact that the same experienced observer performed all the samples observations and contributes significantly to the reduction and control of the confusion variables.

Finally, another limitation was that although we measure marginal discrepancies in different parameters, this issue is not something new, even the technique of manufacturing the crowns on implants; however, the study has an important clinical relevance on all in countries where access to the CAD/CAM CAD technology (computer aided design/computer aided manufacturing) for the manufacture of dental crowns is still scarce.

## 5. Conclusions

In conclusion, according to the results it was found that ZP cement showed the greatest MD after cementation, followed by glass ionomer, while EfZO showed the lowest MD.

## Figures and Tables

**Table 1:** Comparison of the statistical significance of the marginal discrepancy from the four groups.

Group	Mean	SD	Min	Max	Shapiro–Wilk	Kruskal–Wallis
Coping	19.75	3.33	15.70	26.90	0.053	0.001^*∗*^
EfZO	42.75	7.83	29.20	55.22	0.648	0.001^*∗*^
GI	41.72	9.10	25.22	57.60	0.281	0.001^*∗*^
ZP	53.59	14.21	38.82	85.66	0.032	0.001^*∗*^

Values in mean are presented in *μ*. Coping, control group without any cement; EfZO, eugenol‐free zinc oxide; GI, glass ionomer; ZP, zinc phosphate. ^*∗*^Statistically significant.

**Table 2:** Post hoc Bonferroni test of the marginal discrepancy from the four groups.

Group	Coping	EfZO	GI
EfZO	(19.75/42.75)^*∗*^ 0.001		
GI	(19.75/41.72)^*∗*^ 0.001	(42.75/41.72) 1.000	
ZP	(19.75/53.59)^*∗*^ 0.001	(42.75/53.59)^*∗*^ 0.001	(41.72/53.59)^*∗*^ 0.001

Values in mean are presented in *μ*. Coping, control group without any cement; EfZO, eugenol‐free zinc oxide; GI, glass ionomer; ZP, zinc phosphate. ^*∗*^Statistically significant.

## Data Availability

The data used to support the findings of this study are available from the corresponding author upon request.
